# Case Report: Susceptibility to viral infections and secondary hemophagocytic lymphohistiocytosis responsive to intravenous immunoglobulin as primary manifestations of adenosine deaminase 2 deficiency

**DOI:** 10.3389/fimmu.2022.937108

**Published:** 2022-09-09

**Authors:** Enrico Drago, Francesca Garbarino, Sara Signa, Alice Grossi, Francesca Schena, Federica Penco, Elettra Santori, Fabio Candotti, Kaan Boztug, Stefano Volpi, Marco Gattorno, Roberta Caorsi

**Affiliations:** ^1^ Department of Neuroscience, Rehabilitation, Ophthalmology, Genetics, Maternal and Child Health (DINOGMI), University of Genoa, Genova, Italy; ^2^ Center for Autoinflammatory Diseases and Immunodeficiencies, Istituto di Ricovero e Cura a Carattere Scientifico (IRCCS) Istituto Giannina Gaslini, Genova, Italy; ^3^ Unità Operativa Semplice Dipartimentale (UOSD) Laboratory of Genetics and Genomics of Rare Diseases, Istituto di Ricovero e Cura a Carattere Scientifico (IRCCS) Istituto Giannina Gaslini, Genova, Italy; ^4^ Division of Immunology and Allergy, Centre Hospitalier Universitaire Vaudois CHUV, Lausanne, Switzerland; ^5^ Ludwig Boltzmann Institute for Rare and Undiagnosed Diseases, Vienna, Austria; ^6^ St. Anna Children’s Cancer Research Institute, Vienna, Austria; ^7^ CeMM Research Center for Molecular Medicine of the Austrian Academy of Sciences, Vienna, Austria; ^8^ Medical University of Vienna, Department of Pediatrics and Adolescent Medicine, Vienna, Austria; ^9^ St. Anna Children’s Hospital, Vienna, Austria

**Keywords:** dada2, hemophagocitic lymphohistiocytosis, viral infection, therapy, immunoglobulin

## Abstract

Deficiency of adenosine deaminase 2 (DADA2) is an autosomal recessive disease associated with a highly variable clinical presentation, including systemic vasculitis, immunodeficiency, and cytopenia. We report a case of a 16-year-old girl affected by recurrent viral infections [including cytomegalovirus (CMV)-related hepatitis and measles vaccine virus-associated manifestations] and persistent inflammation, which occurred after Parvovirus infection and complicated by secondary hemophagocytic lymphohistiocytosis (HLH). HLH’s first episode presented at 6 years of age and was preceded by persistent fever and arthralgia with evidence of Parvovirus B19 infection. The episode responded to intravenous steroids but relapsed during steroids tapering. High-dose intravenous immunoglobulin (IVIG) helped manage her clinical symptoms and systemic inflammation. The frequency of IVIG administration and the dosage were progressively reduced. At the age of 9, she experienced varicella zoster virus (VZV) reactivation followed by the recurrence of the inflammatory phenotype complicated by HLH with neurological involvement. Again, high-dose steroids and monthly IVIG resulted in a quick response. Targeted next-generation sequencing (NGS) for autoinflammatory diseases and immunodeficiencies revealed the homozygous Leu183Pro ADA2 mutation, which was confirmed by Sanger analysis. ADA2 enzymatic test showed a complete loss of ADA2 activity. For about 3 years, IVIG alone was completely effective in preventing flares of inflammation and neurological manifestations. Anti-TNF treatment was started at the age of 13 for the appearance of recurrent genital ulcers, with a complete response. This case further expands the clinical spectrum of DADA2 and emphasizes the importance of extensive genetic testing in clinical phenotypes characterized by persistent unspecific inflammatory syndromes. The use of high doses of IVIG might represent a possible effective immune modulator, especially in combination with anti-TNF treatment.

## Introduction

Deficiency of adenosine deaminase 2 (DADA2) is a monogenic autoinflammatory disease with autosomal recessive inheritance characterized by pleiotropic clinical manifestations (1, 2). The clinical spectrum of this condition has expanded considerably, ranging from multisystemic inflammation-related symptoms (fever, systemic vasculitis, childhood-onset ischemic and hemorrhagic stroke, etc.) to hematologic abnormalities and immunodeficiency (pure red cell aplasia, neutropenia, lymphopenia, hypogammaglobulinemia, recurrent infections, etc.) (3, 4).

ADA2 is a low-affinity enzyme that catalyzes the deamination of adenosine to inosine, and 2’-deoxyadenosine to 2’-deoxyinosine. It plays a key role in the regulation of the purinergic signaling pathway (5, 6). Activated monocytes, macrophages, and dendritic cells are thought to be the major source of ADA2 in plasma and tissues, where the enzyme also acts like a growth factor involved in endothelial and hematopoietic cell development (7). Changes in the adenosine metabolism pathways result in a complex picture of endothelial cell instability, dysregulation of neutrophil extracellular trap formation (NETosis) (8), and prominence of pro-inflammatory M1 macrophages with an increase of TNF-α production (2).

The assessment of the enzymatic activity can be of help in addressing the diagnosis, which is confirmed by the presence of biallelic mutations in the encoding gene adenosine deaminase 2 (ADA2), located on chromosome 22q11.1.

Although there are currently no shared guidelines for therapy, anti-TNF agents have been shown to be largely effective in controlling fever episodes and vasculopathy, and preventing strokes (9–11). Non-responsive patients may be candidates for hematopoietic stem cell transplantation (HSCT), particularly those with a phenotype characterized by immunodeficiency, cytopenia, and bone marrow failure (12).

Here, we present the case of a 16-year-old girl of Vietnamese origin with a non-typical clinical presentation, characterized by recurrent viral infections and persistent inflammation complicated by the occurrence of secondary HLH.

## Case description

### Clinical course

A 6-year-old female patient was referred to our clinic for nonspecific systemic inflammatory symptoms represented by high persistent fever and arthralgia.

The girl was born in Vietnam and was adopted at the age of 7 months, with unknown family history. In the first years of life, she complained with recurrent respiratory infections and impaired immunological response to viruses. At the age of 9 months, the patient suffered from post-neonatal cytomegalovirus (CMV) infection with hepatic involvement, and at the age of 2 years, she developed measles vaccine virus-associated manifestations (fever, mucositis, and maculopapular eruption).

At admission, the patient presented with persistent fever (with temperatures up to 39.5°C), an increase in C-reactive protein (CRP), and no apparent source of active infection. Autoimmunity screening (ANA, anti-dsDNA, ANCA, and ENA) showed negative results. ASLO, IgA, IgM, IgG, and mevalonic acid dosages on urine were normal. Parvovirus B19 active phase infection was evident (PCR-DNA+, IgM+, and IgG+). Soon after the onset of the systemic inflammatory symptoms, the patient showed the appearance of a maculopapular rash on the face that spread to the trunk and limbs, sparing her hands and feet. The blood tests revealed pancytopenia, increased CRP, hyperferritinemia, hypertransaminasemia, hypertriglyceridemia, and increased LDH (**Table 1**). Bone marrow aspiration confirmed the suspect of hemophagocytic lymphohistiocytosis (HLH). Treatment with intravenous steroids (methylprednisolone 2 mg/kg/day) was started, with prompt response. Chronic low-grade Parvovirus B19 replication was detected on blood samples by PCR for months. During steroids tapering, fever and systemic inflammation reappeared. To overcome steroid dependency, anti-IL-1 treatment (anakinra) was started, without a clear benefit.

Due to the suspicion of an immunological defect characterized by an aberrant response to viral infections, high-dose IVIG (2 g/kg every month) was started, which allowed to control both the clinical sympotms and the inflammatory markers and therfore to reduce steroidal treatment (**Figure 2**). The frequency of IVIG administration was progressively reduced to every 4 months due to persistent wellbeing.

**Figure 1:**
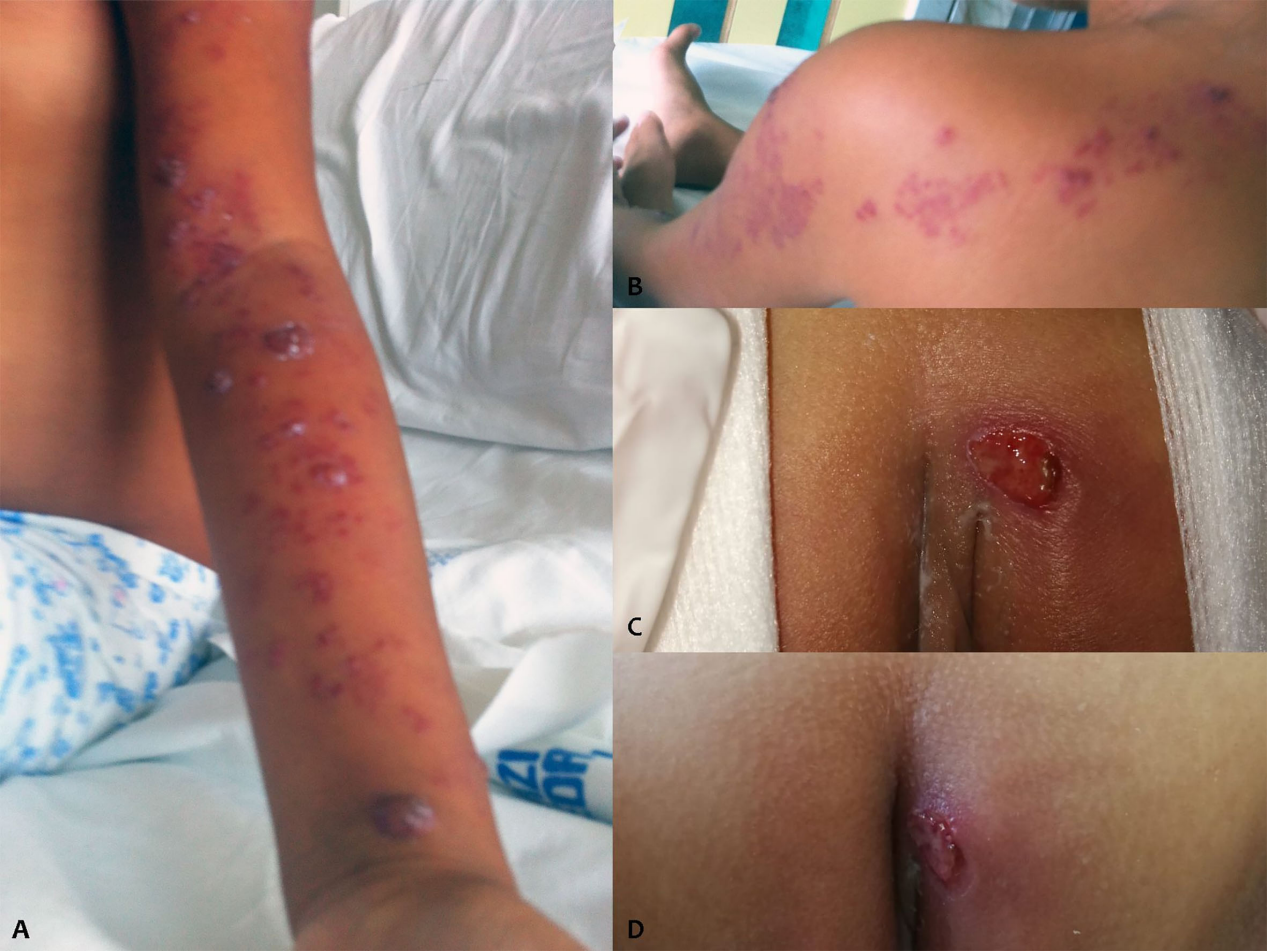
VZV reactivation **(A, B)** and vulvar ulcers **(C, D)** of the patient.

At the age of 8 years, the patient experienced the metameric appearance of vesicles on the whole left upper limb up to the back, with interruption at midline level, suggestive of VZV reactivation (**Figure 1**). The detection of VZV DNA through polymerase chain reaction (PCR) in the vesicles confirmed the diagnosis. Intravenous therapy with acyclovir for 7 days was effective. However, in the following days, the girl started to experience persistent high fever with elevation of acute-phase reactants, promptly responsive to high-dose IVIG infusion.

After about 7 months, due to persistent wellbeing, the dosage of immunoglobulins was reduced and a substitutive regimen was used (400 mg/kg every 28 days). A few months later, the girl experienced a recurrence of the inflammatory phenotype presenting with high fever not responding to antipyretics, followed by the appearance of irritability and lethargy. An electroencephalogram was performed, consistent with encephalitis. Considering these findings and the blood tests (**Table 1**), the diagnosis of HLH with neurological involvement was established. Peripheral blood type I interferon signature was elevated (**Supplementary Figure 1**). Both clinical manifestations and laboratory parameters were responsive to treatment with high-dose intravenous steroids and immunoglobulins (2 g/kg). A follow-up cerebral magnetic resonance imaging (MRI) showed an isolated white matter gliotic area in the left centrum semiovale. During steroids tapering, the patient experienced CMV reactivation, treated by anticipating the scheduled immunoglobulin administration, and afterwards an episode of palpebral ptosis, spontaneously solved, without any evidence of new lesions on brain MRI.

**Table 1:** Main blood tests of the patient at the time of the two episodes of hemophagocytic lymphohistiocytosis (HLH).

	Reference range	First episode of sHLH (May 2012, other center)	Second episodes of sHLH (May 2015)
White cell count (per mm^3^)	3.9–5.6	1.99	2.72
Neutrophils (per mm^3^)	2.1–6.43		1.84
Lymphocytes (per mm^3^)	1.37–6.81		0.78
Monocytes (per mm^3^)	0.24–0.71		0.03
Hemoglobin (g/dl)	11.5–16.5	8.8	11.4
Platelet count (per mm^3^)	150–450	125	124
C-reactive Protein (mg/dl)	0–0.46	2.1	5.56
Erythrocyte sedimentation rate (mm/h)	1–12		18
Serum amyloid A (mg/L)	0–6.4		121
Triglycerides (mg/dl)	30–160		254
Aspartate aminotransferase (U/L)	0–35		450
Alanine aminotransferase (U/L)	0–35		590
Lactate dehydrogenase (U/L)	84–480	1,195	2,524
Ferritin (ng/ml)	20–200	1,164	1,773
Immunoglobulin A (mg/dl)	34–305	31	84
Immunoglobulin M (mg/dl)	500–1,560	448	1,041
Immunoglobulin G (mg/dl)	25–210	29	28
IgG anti-diphtheria (UI/ml)		0.2	
IgG anti-tetanus (UI/ml)		0.45	
PCR-CMV		Negative	Negative
PCR-EBV		Negative	Negative
EBV-IgM	0–0.9	<0.8	<0.8
PCR-Parvovirus B19		Positive	Slightly positive
Parvovirus B19—IgM	0–17	35	negative
Parvovirus B19—IgG	0–11.5	20	62.1
Bone marrow aspiration		Macrophages with hemophagocytosis	
CD3+ (n/mmc)	1,400–2,000		651.3
CD3+ (%)	66–76		83.5
CD3+ CD4+ (n/mmc)	700–1100		429
CD3+ CD4+ (%)	33–41		55
CD3+ CD8+ (n/mmc)	600–900		187.2
CD3+ CD8+ (%)	27–35		24
CD3+ HLA-DR+ %	9.5–17		5.7
CD19+ (n/mmc)	300–500		106.08
CD19+ (%)	12–22		13.6
CD16+ CD56+ CD3- (n/mmc)	200–300		17.6
CD16+ CD56+ CD3- (%)	9–16		2.2
% CD3+ TCRα/β CD4- CD8- (DNTc) (%)	<2.5%		1.9

**Figure 2:**
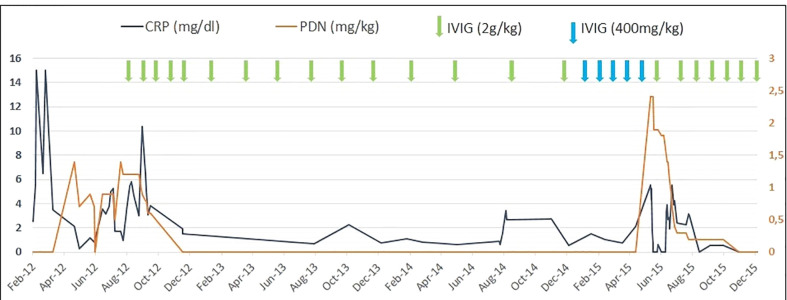
C-reactive protein (CRP) values, dosage of steroidal treatment (prednisone equivalent), and immunoglobulin infusions in the patient during the follow-up period.

After steroids suspension, monthly high-dose (2 g/kg) IVIG maintained prolonged clinical remission for 3 years. (**Figure 2**)

### Immunological and genetic assessment

A genetic and functional study ruled out the presence of immunodeficiencies associated with defects of lymphocyte cytotoxicity compatible with a primary HLH. Enzymatic tests excluded adenosine deaminase 1 (ADA1) and purine nucleoside phosphorylase (PNP) deficiency.

The study of lymphocytic subpopulations performed several times during the clinical course of the disease showed an absolute and relative reduction of NK lymphocytes (**Table 1**) with normal degranulation test and perforin expression; moreover, a mild increase in CD4 and CD8 double-negative (DN) lymphocytes was evidenced.

Normality of Fas-mediated apoptosis and the lack of diagnostic criteria ruled out an autoimmune lympho-proliferative syndrome (ALPS). Mild hypogammaglobulinemia prior to initiation of IVIG therapy was noted. The humoral immune responses for the tetanus–diphtheria vaccine showed a good response and the lymphocytes’ proliferation test was normal.

**Figure 3:**
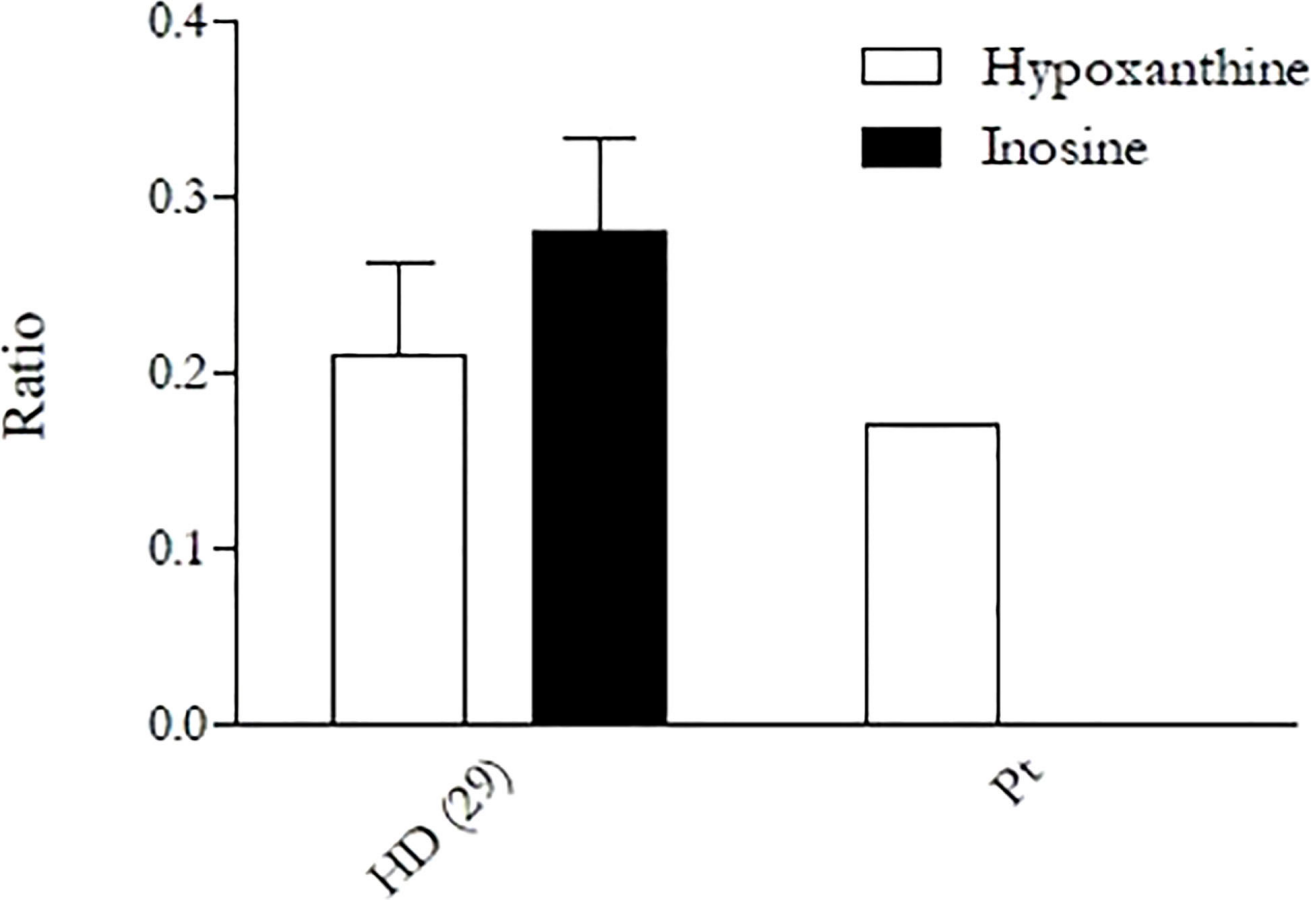
Enzymatic activity of ADA2 in the patient and a healthy donor performed by semiquantitative assay. Activity was assessed in primary monocytes, which were then cultured in PBS in the presence of exogenous adenosine (15 µM), with or without the ADA1 inhibitor EHNA (30 µM). After 4 h of incubation, supernatants were collected, and the activity was evaluated through the measurement of the adenosine-derived products (inosine and hypoxanthine) in high-performance liquid chromatography. The “Ratio” on the *y*-axis is given by the ratio of inosine and hypoxanthine found in the supernatant to the remaining adenosine. The result is again divided by the amount of protein obtained from the cell lysate in order to normalize the result and make it independent from monocyte counts.

Given the complex clinical picture, a large next-generation sequencing (NGS) diagnostic panel for autoinflammatory diseases and immunodeficiencies was performed at St. Anna Children’s Hospital in Vienna. The panel detected the presence of the novel Leu183Pro (c. 548 T>C, RefSeq: NM_001282225.2) homozygous missense mutation in ADA2 gene, confirmed by Sanger analysis (**Supplementary Figure 2**), and the novel IRAK1 A15G homozygous missense mutation. *In silico* tools (PolyPhen2 and CADD) predicted the *ADA2* variant to be “probably damaging” (PolyPhen score: 0.996 and CADD score: 23.4) and the IRAK1 mutation to be benign (PolyPhen score: 0.005 and CADD score: 22.4). ADA2 mutation is currently registered on Varsome as Uncertain Significance (PM1 Moderate - PM2 Supporting - BP4 Supporting) according to ACMG guidelines. In order to confirm the pathogenetic role of the mutation, ADA2 semiquantitative enzymatic activity test was performed, as previously described (11). The test showed a decrease in the production of hypoxanthine and a complete absence of inosine formation by the patient’s PBMCs, revealing a complete loss of ADA2 activity (**Figure 3**). Plasma ADA2 enzymatic activity was 5.6 mU/g protein (control range 58–271) when measured in extracts of dried plasma spots by the Hershfield lab at Duke University, as described by Ben Ami et al. (13). We therefore concluded that the clinical presentation of the patient was caused by ADA2 mutation.

### Follow-up and current treatment

After DADA2 diagnostic confirmation, the parents were informed about the indication to anti-TNF treatment related to the risk of strokes (14, 15). However, due to the previous history of repeated viral infections, the parents refused this treatment. Therefore, treatment with high-dose IVIG was maintained and progressively reduced up to every 42 days, without recurrence of inflammatory symptoms.

At the age of 13, after about 3 years of wellbeing, recurrent multiple vulvar ulcers appeared (**Figure 1C**). By virtue of the negative microbiological findings (swab for viruses and bacteria, including HSV1/2), poor response of the lesions to antibiotic and antiviral therapy, the partial response to the application of topical steroid, and the characteristic of the lesions (cutaneous essudation and infiltration), we hypothesized the vasculitic nature of the ulcers. A skin biopsy revealed the presence of signs of vasculitis. Therefore, treatment with etanercept at a dosage of 0.8 mg/kg/week was proposed to the parents and finally accepted, which resulted in a quick resolution of skin lesions (**Figure 1D**) and absence of subsequent relapses of genital ulcers. The dosage of the immunoglobulins was reduced, moving to a sustitutive regimen, and the frequency of administration was progressively reduced, due to persistent wellbeing. Periodic brain MRIs excluded any neurological complication. No major viral or bacterial infections were observed.

## Discussion

The current report widens the pleiotropic clinical spectrum associated with DADA2 to a persistent unspecific inflammatory syndrome complicated by virus-induced HLH. Given the unusual phenotype, diagnosis could be achieved thanks to an extensive genetic study (NGS) that detected the Leu183Pro (c. 548 T>C) homozygous mutation, previously not described in the literature. The lack of the enzymatic activity allowed us to confirm the diagnosis of DADA2.

To the best of our knowledge, no other reports of an isolated hyperinflammatory syndrome complicated by secondary HLH in DADA2 patients are present so far in the literature. Moreover, this is the first case in which a complete and sustained clinical response to high-dose IVIG regimen alone was reported. These clinical and therapeutic aspects might have a possible multifactorial origin, such as the presence of other recessive gene variants (due to the high probability of parental consanguinity) or epigenetic factors. A possible candidate is IRAK1: it encodes for a serine/threonine protein kinase involved in TLR signaling (16). Interestingly, IRAK1 is highly expressed in NK cells, which were mildly reduced in our patient. Recruitment and activation of IRAK1 leads to downstream recruitment of TRAF6, and both IRAK1 and TRAF6 are thought to be important in NF-KB signaling, which is relevant to HLH pathogenesis, and to IFNα induction, crucial in host antiviral defense (16). Although the mutation was predicted *in silico* as benign, a functional assay to verify pathogenicity is lacking.

Purinergic signaling alteration in ADA2 deficiency has been demonstrated to cause an inflammatory microenvironment due to high adenosine concentration on surfaces of immune cells, leading to activation of R-A_2B,_ a purine receptor with a broad pro-inflammatory action mediating, among others, the release of IL-6 from activated monocytes and TNF-alpha from neutrophils *in vitro* (7). Moreover it has been demonstrated that DADA2 patients show a neutrophil pattern characterized by low-density granulocytes (LDGs) with a tendency for neutrophil extracellular trap formation (NETosis) mediated by adenosine (A_1_ and A_3_ receptors), contributing to the amplification of inflammation and endothelial instability (8).

In DADA2 patients, the monocyte–macrophage cell line has been advocated to have a pivotal role on inflammation amplification, as suggested by the skew towards M1 pro-inflammatory phenotype (11), the association with activated neutrophils in inflamed tissue specimens (8), and the interaction with lymphocytes in TH1 response, which could cause a constitutive activation of the M1 macrophages and the release of high amounts of pro-inflammatory cytokines (TNFalpha, IFN gamma, IL-12, and IL-18).

Recently, ADA2 activity levels have been described to be elevated in several chronic infectious and inflammatory diseases (17), and it has been advocated as a marker of macrophage activation syndrome (MAS), a secondary HLH associated with rheumatic illnesses, playing a still uncertain immunomodulating role within these conditions characterized by hypercytokinemia (18).

Besides the present case, there are two other reports of secondary HLH associated with DADA2 (19, 20), suggesting that the hyperactivation of proinflammatory macrophages itself is rarely sufficient to provoke the hypercytokinemic syndrome leading to secondary HLH. In our patient, the recurrence of the disease was frequently accompanied by a viral infection or reactivation, which is a known trigger for macrophage activation and TH1 immune response, possibly causing a second strong inflammatory signal in the context of an already inflamed subset. With regard to these speculations, there have been reports on an elevated Interferon type I Signature (IS) in DADA2 patients, as observed in our patient (21); however, the mechanisms leading to the hyperactivation of IFN Type I pathway in these patients have not been completely clarified so far.

Our patient presented a clear predisposition to viral infection, with a severe disease course, or reactivation (CMV, Parvovirus B19, VZV), even in the absence of clear immunological features suggestive of immunodeficiency. In fact, apart from a mild reduction in the count of NK cells and of the plasmatic levels of Ig, without evidence of bacterial respiratory infection (such as sinusitis, otitis, and pneumonia), as typically observed in hypogammaglobulinemia, no other findings suggestive of immunodeficiency were detected. The pathogenic mechanism of the immunological defect of DADA2 is widely unknown, apart from the possible role of growth or differentiating factor for several immune cells, as demonstrated by the fact that ADA2 shares high sequence similarity with adenosine deaminase growth factors (ADGFs) (3, 17). The lack of activity is linked to a maturation blockade of hematopoietic progenitors and to several immune cell dysfunctions: on CD16+ monocytes, it is associated with M1 macrophage polarization (1, 11), and on B lymphocytes, it causes a lack of differentiation as demonstrated by memory B cell depletion and hypogammaglobulinemia (22). In addition, a decrease in NK and cytotoxic lymphocytes has been found to be common in many DADA2 patients. Finally, even if the exact mechanism is still widely unknown, *in vitro* data suggested that ADA2 binds specific receptors on T-cell surfaces (3, 17, 23).

The heterogeneous phenotypic manifestations of ADA2 deficiency have brought about several attempts to find out a genotype–phenotype correlation, but this seems to be complicated by epigenetics, environmental factors, and probable incomplete penetrance of mutations, leading to variable clinical manifestations in family members with identical genotype (4). Recently, it has been proposed that mutations that are most detrimental to protein function, as measured by residual ADA2 activity, are more likely to correlate with severe hematologic involvement (24), suggesting that only a small amount of ADA2 is required to maintain normal hematopoiesis.

In the present case, genetic analysis was made difficult by the absence of parental genotype, the patient being adopted, and was further complicated by the atypical clinical picture for DADA2; therefore, NGS has been crucial for the diagnosis and allowed us to identify a novel homozygous missense mutation in the ADA2 gene, Leu183Pro (c. 548 T>C), causing a complete loss of function of ADA2 on the enzymatic assay. Despite the complete lack of enzymatic activity, neither cytopenia nor bone marrow failure was evident: conversely, the patient showed an unusual hyperinflammatory phenotype with immune dysregulation. Further studies are needed to understand if this novel mutation could determine such a unique clinical presentation, or this may be due to the combination with mutations of other genes involved in the immune response, or other epigenetic and environmental factors.

The current literature on DADA2 indicates that TNF inhibitors are successful in rescuing the acute inflammatory phenotype, preventing inflammatory flares and, consequently, the occurrence of life-threatening events (strokes, GI perforations, etc.). However, this drug is generally described to have a minor impact on the severe hematological complications, usually requiring HSCT. Anti-TNF treatment represents a pivotal therapeutic strategy whenever the hematological manifestations are associated with a severe inflammatory phenotype with high risk of strokes. However, in these conditions, the risk of severe infection might represent a relevant limitation in the management of patients. Other immunomodulatory drugs have been used in DADA2, with inconstant and often incomplete success (11). In the present case, therapeutic strategy was initially guided by the goal of controlling the hyperinflammatory phenotype, being unaware of the diagnosis; the use of high-dose steroids and immunoglobulins was effective to treat HLH episodes and to reduce the occurrence of viral infections, while high doses of immunoglobulins alone allowed a complete control of the underlying systemic inflammation. There are no other reports of the successful use of high-dose immunoglobulins alone as an anti-inflammatory strategy in both induction and maintenance of remission in DADA2 patients, which is described for the acute phase in a few cases only, in association with other immunomodulants, but found to be insufficient alone for long-term remission (12, 25). In our case, any attempt to reduce the dosage or frequency of administration of immunoglobulins less than 2 g/kg every 6 weeks was followed by a relapse in inflammation and viral reactivations/infections, suggesting a crucial role of high-dose immunoglobulins in controlling systemic inflammation in this patient.

IVIGs are known to be used as prophylaxis or treatment for infectious diseases, particularly in viral infections. IVIGs are also known to be effective in inflammatory conditions associated with endothelial activation and/or microangiopathy, such as Kawasaki disease and dermatomyositis. The anti-inflammatory mechanism of high-dose immunoglobulin is complex and largely unknown. It has been demonstrated that IVIGs have an inhibitory effect on antigen-specific T-cell proliferation (25); moreover, the IVIG Fc binding to Fc receptors on macrophage can deactivate phagocytosis (26). These two mechanisms can explain the role of this treatment in controlling virus-induced HLH. The immunomodulatory action of IVIG, able to reduce the level of pro-inflammatory cytokines (such as TNFα) (27) and to inhibit the endothelial activation (28), can partially explain the role of this treatment in the prevention of the inflammatory and vascular manifestation of the disease. Interestingly, another role that IVIG might have played in this patient is shown by the induction of multiple phenotypic and functional changes in NK cells, mainly promoting resolution of inflammation (29).

As previously described (11), treatment with TNF inhibitors not only was effective to maintain remission of the inflammatory phenotype, but also allowed a progressive reduction of IVIG replacement, since the plasmatic levels of immunoglobulin reached the normal range and the patient did not complain with recurrent infections after the beginning of biological treatment. The possible mechanism by which anti-TNF treatment may lead to a restoration of normal level of plasmatic immunoglobulin in DADA2 is unknown.

## Conclusion

This case further expands the clinical spectrum of DADA2 and emphasizes the importance of extensive genetic testing (NGS and/or WES) in unraveling unusual phenotypes of already known inflammatory syndromes. The availability of different enzymatic tests (13, 30–32) sepcificity, has simplified the diagnosis workup of this condition, which needs to be ruled out in case of persistent inflammation, associated or not with signs of immunodeficiency and vasculopathy.

Even if TNF inhibitors represent so far the treatment of choice of patients with a predominant inflammatory phenotype, the use of high-dose IVIG might offer a possible further support in patients with a severe hematological phenotype with high risk of infections.

## Data availability statement

The original contributions presented in the study are included in the article/**Supplementary Material**. Further inquiries can be directed to the corresponding author.

## Ethics statement

Written informed consent was obtained from the minor(s)’ legal guardian/next of kin for the publication of any potentially identifiable images or data included in this article.

## Author contributions

Authors contribution statement: ED., FG and SS collected the clinical data of the patient. ED and FG drafted the first version of the paper. FS and FP performed the functional tests of ADA2 activity. ES and FC performed the IF signature test, KB performed the NGS panel and AG the sanger confirmation of the detected mutation. SV, MC and RC drafted the final version of the paper RC coordinated the data collection. All authors contributed to the article and approved the submitted version.

## Conflict of interest

The authors declare that the research was conducted in the absence of any commercial or financial relationships that could be construed as a potential conflict of interest.

## Publisher’s note

All claims expressed in this article are solely those of the authors and do not necessarily represent those of their affiliated organizations, or those of the publisher, the editors and the reviewers. Any product that may be evaluated in this article, or claim that may be made by its manufacturer, is not guaranteed or endorsed by the publisher.

## Supplementary material

Supplementary Figure 1 Positive peripheral blood type I interferon signature during second episode of HLH.

Supplementary Figure 2 Chromatogram of sanger sequencing of the patient (bottom) and a control (top)
