# Supplementary figures and images for: Case Report: Susceptibility to viral infections and secondary hemophagocytic lymphohistiocytosis responsive to intravenous immunoglobulin as primary manifestations of adenosine deaminase 2 deficiency
Source: Front Immunol. 2022 Sep 9;13:937108. doi: 10.3389/fimmu.2022.937108 (PMC9503826; doi:10.3389/fimmu.2022.937108)

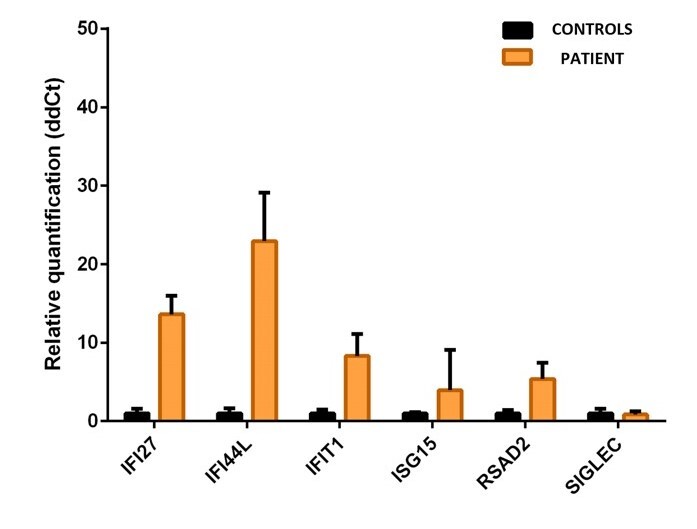

Supplement: Supplementary Figure 1 — Positive peripheral blood type I interferon signature during second episode of HLH. [file Image_1.tiff]

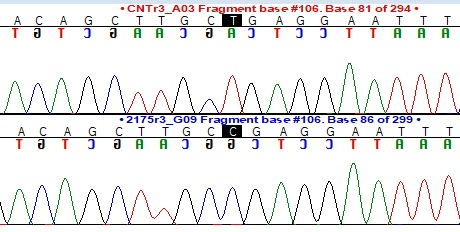

Supplement: Supplementary Figure 2 — Chromatogram of sanger sequencing of the patient (bottom) and a control (top) [file Image_2.tif]
